# Salmonella Peritonitis Secondary to Gastroduodenal Perforation: An Unusual Presentation of Enteric Fever

**DOI:** 10.7759/cureus.65090

**Published:** 2024-07-22

**Authors:** Lavanya Balaji, Subbalakshmi Easwaran, Jayakumar Subramaniam

**Affiliations:** 1 Department of Microbiology, Saveetha Medical College and Hospitals, Saveetha Institute of Medical and Technical Sciences, Saveetha University, Chennai, IND

**Keywords:** salmonella peritonitis, atypical salmonella, duodenal perforation, salmonella enterica serovar paratyphi a, enteric fever (typhoid fever)

## Abstract

*Salmonella*-induced peritonitis, secondary to spontaneous gastrointestinal perforation, is a rare but potentially life-threatening condition. We present a case of a 62-year-old female with a history of systemic hypertension, who presented with diffuse abdominal pain and altered bowel habits. Initial evaluation suggested acute gastroenteritis, but worsening symptoms led to emergent exploratory laparotomy, revealing a gastric/duodenal perforation. Peritoneal fluid analysis and culture confirmed *Salmonella* Paratyphi A infection. The patient underwent an emergency laparotomy with omental patch repair and received intravenous ceftriaxone, leading to a full recovery. This case underscores the importance of considering *Salmonella* infection in the differential diagnosis of peritonitis, prompt surgical intervention, and appropriate antimicrobial therapy for optimal management and outcomes. Further research on epidemiological trends, host-pathogen interactions, and antibiotic resistance should be explored. Clinical studies should refine diagnostic criteria and treatment protocols, while animal models can aid in understanding pathophysiology and vaccine development for *Salmonella* peritonitis. Public health interventions and environmental studies will enhance prevention and control strategies.

## Introduction

Peritonitis, the inflammation of the peritoneum, is a serious and urgent surgical condition that can be life-threatening. It causes severe abdominal pain and significantly impacts morbidity and mortality, with rates ranging from 10% to 60% in surgical settings [1]. The causes of peritonitis vary depending on geographic locations, local environmental factors, and genetic predisposition. The most common causes are appendicitis and peptic ulcer disease, accounting for about 22% and 11% of cases, respectively. Other causes include gastroduodenal perforations, intestinal volvulus, ruptured abscesses, traumatic bowel perforation, primary or idiopathic peritonitis, tubo-ovarian abscesses, and amoebic colonic perforations [2].

*Salmonella*, a gram-negative bacterium, is most commonly associated with gastrointestinal infections [3]. However, it can cause peritonitis by entering the abdominal cavity. While ileal perforation is the common site for *Salmonella* peritonitis, it can also result from gastroduodenal perforation in rare instances. Intestinal perforation occurs when the ulceration and inflammation caused by a typhoidal infection weaken the intestinal wall, resulting in perforation. This perforation allows the contents of the intestine, including bacteria and faecal matter, to leak into the abdominal cavity, leading to peritonitis, a severe and potentially life-threatening condition [4]. Symptoms of peritonitis may include sudden worsening of abdominal pain, distension, fever, altered mental status, and signs of systemic inflammation [5].

Diagnosis involves analysing the ascitic fluid, which may show an elevated white blood cell count with a predominance of neutrophils and positive culture results for *Salmonella* [6]. Treatment involves surgical repair of the perforation and antimicrobial therapy to address the underlying typhoidal infection and prevent the further spread of bacteria throughout the body. The mortality and morbidity rate of peritonitis does not primarily depend on the surgical technique but on the patient's general status, the *Salmonella* bacteria's virulence like Type III secretion system (T3SS), and the duration of disease evolution before surgical treatment [7]. Therefore, it is crucial to provide adequate pre-operative management, including aggressive resuscitation and antibiotic therapy. Typhoid fever with perforation is best managed by early surgical intervention. Various surgical options include simple primary closure, primary closure with an omental patch, resection and anastomosis. Faecal fistula, the most common complication of enteric perforation, may occur due to anastomotic dehiscence, reperforation, or a different perforation site. Simple primary closure remains the procedure of choice as it is quick and cost-effective [4,8]. Prevention strategies for high-risk populations include prophylactic antibiotic therapy and measures to reduce bacterial translocation [6]. These strategies are crucial in preventing the onset of peritonitis in vulnerable groups. Overall, while *Salmonella*-induced peritonitis is rare, its diagnosis and management require vigilance to prevent potentially life-threatening outcomes. Here, we report a case of a patient who developed *Salmonella* peritonitis secondary to spontaneous gastroduodenal perforation. The patient presented with severe abdominal pain, distension, fever, and signs of systemic inflammation. Diagnostic tests, including ascitic fluid analysis and imaging studies, confirmed the presence of peritonitis due to a gastroduodenal perforation caused by *Salmonella* infection. The patient underwent surgical repair of the perforation and received appropriate antimicrobial therapy. Following a period of intensive care and supportive management, the patient made a full recovery. This case underscores the importance of recognising the potential for *Salmonella* to cause peritonitis through less common routes, such as gastroduodenal perforation. It highlights the need for prompt, accurate diagnosis, aggressive pre-operative management, and appropriate surgical intervention to achieve favourable outcomes. Effective prevention strategies and awareness are essential in managing and reducing the incidence of this serious condition.

## Case presentation

A female patient in her late 60s presented to the Emergency Department with acute and progressively worsening diffuse abdominal pain over the past three days. She also complained of difficulty in urination and passing loose stools three to four times daily, characterised by a watery consistency and without any presence of blood for the same period. Over the same period, she experienced vomiting, which was non-bilious, with vomitus containing food particles and no blood. The patient has a 15-year history of systemic hypertension and is currently on anti-hypertensive medication. During the initial examination, the patient was found to be febrile (100℉) with vital signs within normal ranges (blood pressure: 100/60 mmHg, pulse rate: 92 beats/minute, 98% oxygen saturation at room air). Systemic examination revealed a soft abdomen with tenderness in the right hypochondriac region, with no guarding or rigidity. The patient was admitted for further evaluation and management of the abdominal pain. Baseline investigations were done, and the results of the laboratory tests are shown in Table 1.

**Table 1:** Routine laboratory investigation reports with reference ranges for this patient.

Parameter	Patient value	Reference value
White blood cell count	17,420 cells/cumm	40-10000 cells/cumm
Neutrophils	93.8%	44.0-72.0%
Lymphocytes	4.8%	18.0-59.0%
Monocytes	1.3%	0.0-12.0%
Eosinophils	0.0%	0.0-10.0%
Basophils	0.3%	0.0-3.0%
Red blood cell count	4.66 million/cumm	3.76-5.50 million/cumm
Haemoglobin	9.8 g/dL	11.3-15.2 g/dL
Haematocrit	32.5%	33.4-44.9%
Platelet count	4.51 lakhs/cumm	1.5-4.1 lakhs/cumm
Absolute neutrophil count	16,390 cells/cumm	2000-7000 cells/cumm
Aspartate aminotransferase (ALT)	14 IU/L	5-50 IU/L
Alanine aminotransferase (AST)	18 IU/L	17-59 IU/L
Alkaline phosphatase level (AKP)	142 IU/L	38-126 U/L
Urea	2.2 g/dL	3.5-5.0 g/dL
Creatinine level	1 mg/dL	0.40-1.10 mg/dL
C-reactive protein level	345 mg/L	<10 mg/L
Erythrocyte sedimentation rate (ESR)	144 mm/hr	0-14 mm/hr

**Figure 1:**
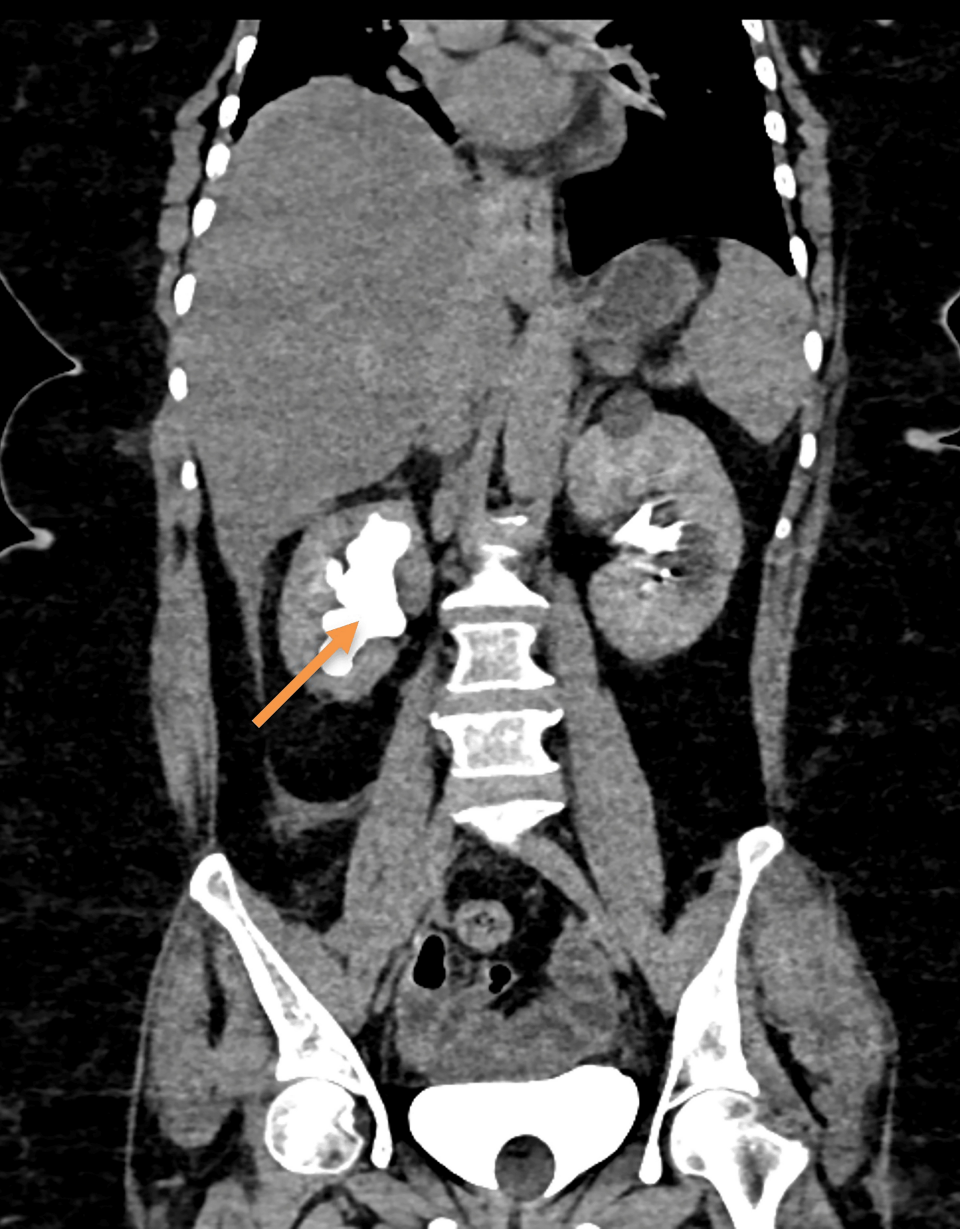
CECT abdomen (coronal section) of the patient. The arrow shows a dilated right pelvicalyceal system (Grade 3 hydronephrosis) with a sudden narrowing of the right proximal ureter. CECT: Contrast-enhanced computed tomography

The results of the lab tests revealed a higher count of white blood cells (17,420 cells/cumm), an increased absolute neutrophil count (16,390 cells/cumm), and a C-reactive protein (CRP) level of 345 mg/L. The patient's lab results indicate an acute bacterial infection, which is supported by elevated levels of white blood cells, neutrophils, absolute neutrophil count, and CRP. Serological tests for human immunodeficiency virus (HIV), hepatitis B surface antigen (HBsAg), and hepatitis C virus (HCV) were negative. The blood culture was sterile, and the stool culture showed normal flora. The Widal test was negative with titres of 1:20 for O and H antibodies. The stool routine was negative for ova and cyst and hanging drop for stool showed no *Vibrio-*like motility. The cell cytotoxic assay was tested negative for* Clostridium difficile*. Imaging with contrast-enhanced computed tomography (CECT) abdomen revealed Grade 3 hydronephrosis with a sudden narrowing of the right proximal ureter, slight inflammation of the perinephric and peri-ureteric fat, thickening of the anterior and posterior para-renal fascia, and mild ascites, suggesting a probable infectious cause, as shown in Figure 1.

**Figure 2:**
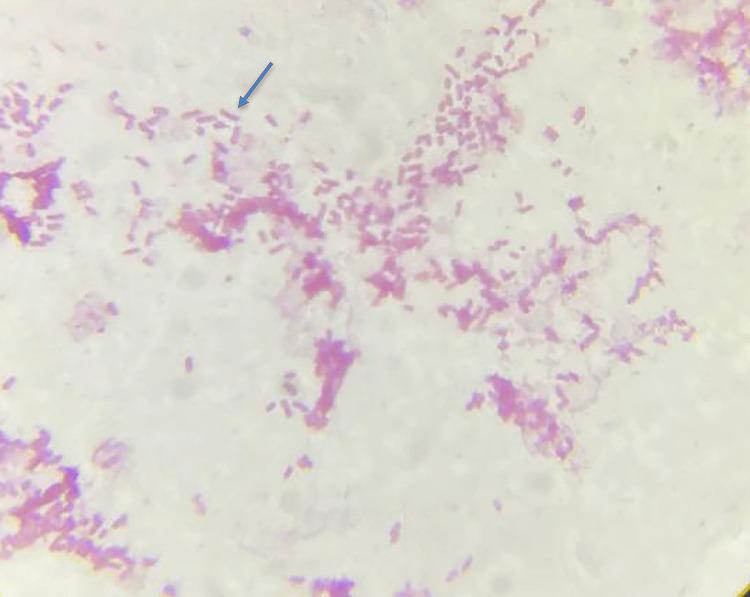
Gram stain of the peritoneal fluid showing occasional inflammatory cells and occasional gram-negative bacilli.

A provisional diagnosis of acute gastroenteritis with Grade 3 hydronephrosis was made, and the patient was empirically started on parenteral piperacillin-tazobactam 4.5 g twice daily and intravenous metronidazole 500 mg once daily because of infection. During the course of the hospital stay, the patient's abdominal pain worsened, resulting in obstipation. A surgical opinion was urgently sought, and the decision was made to perform an emergency explorative laparotomy. Approximately 500-700 mL of seropurulent fluid was drained upon opening the peritoneum. Further exploration revealed a perforation in the gastric/duodenal area. The perforation was successfully sealed with omentum and the transverse colon. The biopsy of the small specimen from the gastric area and the seropurulent fluid was sent for analysis. Histopathological examination of the specimen revealed features of acute suppurative inflammation and was negative for the presence of *Helicobacter pylori* (*H. pylori*) infection. Peritoneal fluid analysis revealed elevated white blood counts (12,000 cells/cumm), abundant neutrophils, and decreased protein levels. The peritoneal fluid was subjected to GeneXpert and culture and sensitivity testing. GeneXpert yielded negative results for *Mycobacterium tuberculosis*, while the gram stain indicated the presence of occasional inflammatory cells with moderate gram-negative bacilli (Figure 2).

**Figure 3:**
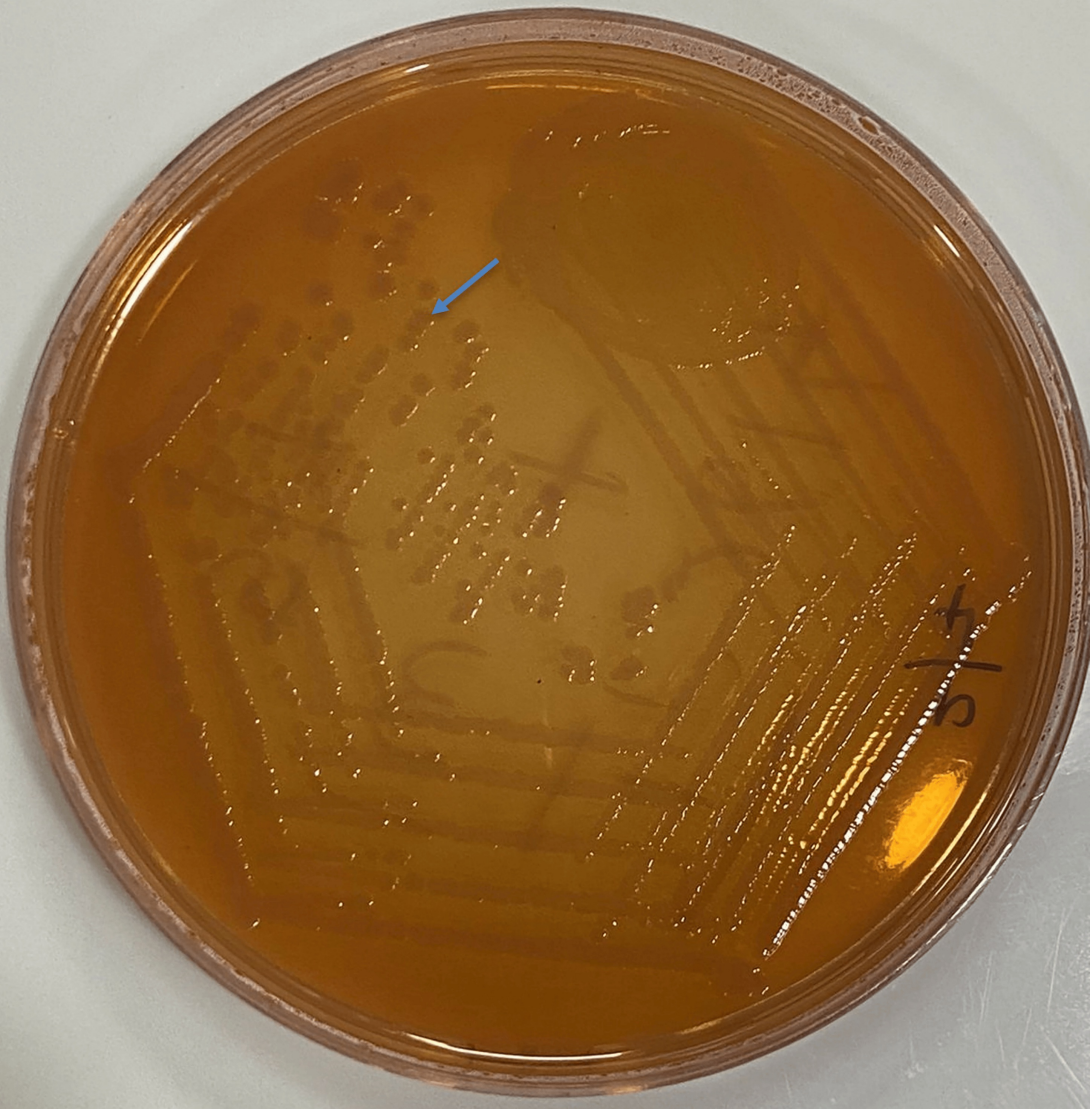
MacConkey agar showing non-lactose fermenting colonies.

Subsequent subculturing on blood, chocolate, and MacConkey agar revealed smooth, greyish-white colonies on blood and chocolate agar, and non-lactose fermenting colonies on MacConkey agar after 24 hours of incubation, as shown in Figure 3.

The organism was identified as *Salmonella* Paratyphi A (*S*. Paratyphi A) using the Vitek2 Compact system (bioMérieux, Inc., Marcy-l'Étoile, France) for automated identification and antibiotic susceptibility testing and further confirmed through sero-agglutination using anti-sera against O and H with antigens of *S*. Typhi and *S*. Paratyphi A and B. Antibiotic susceptibility testing by disk diffusion (DD) on Mueller-Hinton agar (MHA), indicated susceptibility to azithromycin, ceftriaxone, and chloramphenicol. Consequently, the patient was switched to parenteral ceftriaxone 1 g twice daily based on the positive culture results. The patient's hospital course was uneventful, and she fully recovered from peritonitis, experiencing a resolution of abdominal pain. Upon discharge, the patient was transitioned to oral prophylaxis therapy with azithromycin 500 mg once daily. On follow-up after three weeks, the patient was doing well without any complaints or complications.

## Discussion

Intestinal perforation is a significant complication of typhoid fever in the developing world and presents a challenge to surgeons due to its high morbidity and mortality [9]. The development of perforation is unpredictable, and only a few cases have been attributed as secondary to *Salmonella *species. The most common gastrointestinal tract perforations are duodenal and gastric ulcers [10]. These perforations may occur spontaneously or due to trauma, with the majority of spontaneous perforations being caused by peptic ulcer disease [11]. Peptic ulcer disease is primarily caused by *H. pylori *infection and the use of nonsteroidal anti-inflammatory drugs (NSAIDs) [12]. In our patient, *H. pylori *was not found in biopsy specimens, and she had no history of taking NSAIDs or other ulcerogenic drugs. Additionally, she was not using proton pump inhibitors, antibiotics, or bismuth compounds, which could have caused a missed diagnosis of *H. pylori* infection. Therefore, it can be postulated that the peritonitis stemming from a gastroduodenal ulcer in our patient was a consequence of an infection with *S.* Paratyphi A.

The prevalence of typhoidal *Salmonella* causing peritonitis and perforation is rare, accounting for only 0.15% [13]. The reasons behind colonic perforation caused by *Salmonella* infection are not well understood. A study on *Salmonella*-related colonic perforation suggested that bacteria infiltrating Peyer’s patches cause necrosis, which leads to haemorrhage and perforation [14]. Another hypothesis is that an overactive immune system causes an increase in inflammatory cytokine production, leading to the clumping of macrophages and lymphocytes around vascular tissue, ultimately resulting in bowel necrosis [15]. However, the mechanism behind the gastroduodenal perforation in this case is unclear. Typhoid fever with perforation is best managed by early surgical intervention. Various surgical options include primary closure, primary closure with omental patch, resection and anastomosis [16]. In our case, the patient was taken for emergency laparotomy and omental patch repair. Given the positive culture for *Salmonella*, our patient was immediately transitioned to susceptible intravenous antimicrobials to prevent the further spread of infection. The patient's prognosis depends on several factors, including the timing of surgical intervention after perforation, the efficacy of antibiotic treatment, and the quality of supportive care. This includes providing fluids to address hypotension, utilising nasogastric suction, correcting anaemia, and addressing organ failure as needed. For antibiotics, it is recommended to use a combination of cephalosporin, fluoroquinolone, aminoglycosides, metronidazole, or clindamycin [15]. In cases of sepsis, like that of our patient, appropriate hydration and resuscitation are crucial for maintaining hemodynamic stability.

## Conclusions

This case discusses a rare and complex instance of *Salmonella* peritonitis caused by spontaneous gastroduodenal perforation in a patient with acute gastroenteritis and Grade 3 hydronephrosis. The patient initially experienced diffuse abdominal pain, and their symptoms worsened despite antibiotic treatment, highlighting the need for careful diagnosis and timely surgical intervention. Successful patient management involved multiple medical approaches, including emergency laparotomy, drainage of seropurulent fluid, and sealing of the perforation with an omental patch. Identifying* S.* Paratyphi A through culture and sensitivity testing allowed for targeted antimicrobial therapy, which was crucial for the patient's recovery. This case emphasises the importance of considering uncommon causes of peritonitis, such as *Salmonella *infection, particularly in patients exhibiting atypical symptoms and high inflammatory markers. Early recognition, aggressive pre-operative management, and appropriate surgical intervention are key to achieving positive outcomes. Effective prevention strategies and increased awareness are essential in managing and reducing the occurrence of such serious conditions in vulnerable populations.
